# Physical Properties of an Ionic Liquid Composed of Two Water-Soluble Vitamins and Enhanced Skin Permeation of Both Vitamins

**DOI:** 10.3390/pharmaceutics12050427

**Published:** 2020-05-06

**Authors:** Kenji Sugibayashi, Yuya Yoshida, Ryuichiro Suzuki, Kota Yoshizawa, Kenji Mori, Shoko Itakura, Kozo Takayama, Hiroaki Todo

**Affiliations:** 1 Faculty of Pharmacy and Pharmaceutical Sciences, Josai University; 1–1 Keyakidai, Sakado, Saitama 350-0295, Japan; yoshi.yuya.1109@gmail.com (Y.Y.); ryu_suzu@josai.ac.jp (R.S.); gkm2044@josai.ac.jp (K.Y.); sitakura@josai.ac.jp (S.I.); kz-tkym@josai.ac.jp (K.T.); ht-todo@josai.ac.jp (H.T.); 2 Faculty of Pharmaceutical Sciences, Josai International University; 1 Gumyo, Togane, Chiba 283-8555, Japan; kmori@jiu.ac.jp

**Keywords:** liquid crystal, vitamin C, vitamin B6, solubility, skin permeation

## Abstract

A highly viscous substance was prepared by evaporating an ethanol solution containing two hydrophilic vitamins; vitamin C, and vitamin B6. The viscous substance and physical mixture of the two vitamins were tested using a differential scanning calorimeter and an X-ray diffractometer. The highly viscous substance was found to be a liquid crystal (LC) made of these two hydrophilic vitamins. Determination by proton nuclear magnetic resonance measurement suggested that intramolecular hydrogen bonding in vitamin B6 was eliminated by the LC formation. This LC compound showed high solubility in 1,3-butanediol (almost 87%). Much higher skin permeation of both vitamin C and B6 was also observed from the LC compound than that from the physical mixture. The present LC compound containing vitamin C and vitamin B6 may be useful for pharmaceutical and cosmeceutical applications.

## 1. Introduction

In general, a salt composed of a strong acid and a weak base becomes a crystal (solid), whereas some organic salts composed of a weak Lewis acid and a weak Lewis base become a liquid at room temperature. These substances are called liquid electrolytes, ionic melts, ionic fluids, fused salts, liquid salts, or ionic glasses to differentiate them from ordinary liquids. We use the term “ionic liquid (IL)” for these substances in this article [1].

One of the earliest truly room temperature-ILs was ethylammonium nitrate (C_2_H_5_)NH_3_NO_3_ (m.p. 12 °C), reported in 1914 by Paul Walden [2]. However, no active research on ILs was carried out until late in the 20th century. Recently, ILs have started to attract attention as “designer solvents” because they have various functions through adjusting the physical properties of the cationic and anionic species of their constituents [3]. Many commercial applications have already been considered.

In the field of pharmaceutical sciences, IL forms of many pharmaceuticals have been investigated. Combining a pharmaceutically active cation with a pharmaceutically active anion leads to a dual active IL, in which the actions of the two drugs are combined [4,5]. ILs can extract specific active compounds from plants for pharmaceutical, nutritional, and cosmetic applications, such as the antimalarial drug artemisinin from the plant *Artemisia annua* [6]. Improvement of the gastrointestinal absorption of drugs by IL conversion [7,8,9] and improvement of the solubility of poorly soluble drugs using ILs [10,11] have been actively studied. Regarding enhancement of the percutaneous absorption of drugs examined in his study, it has been reported that skin permeation of drugs was enhanced by IL formation [12,13,14,15,16,17] and IL micelle formation as well [18,19]. However, these studies using ILs focused only on improvement of the skin permeability of lipophilic drugs with relatively high skin permeation properties [20,21,22], and few studies have been done to increase the skin permeation of hydrophilic drugs with low skin permeability. Furthermore, these ILs aimed at improving skin permeability, but there have been no reports that the permeability of a plurality of constituent drugs has been improved.

**Figure 1:**
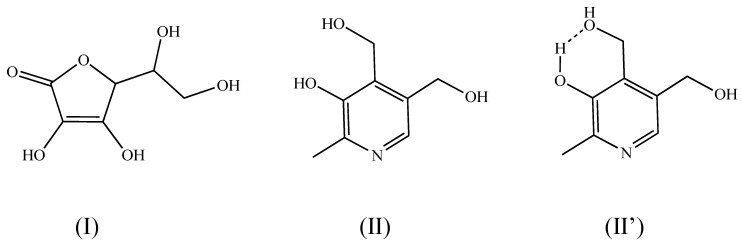
Chemical structure of L-ascorbic acid (VC) (**I**) and pyridoxine (VB6) without (**II**) and with intramolecular hydrogen bond (**II’**).

L-ascorbic acid (VC) (acidic compound; *M.W.* 176.12; log (*K_ow_* − 2.15)) (I in Figure 1) is known as a representative compound with low skin permeability due to its high polarity. Several methods have already been studied for promoting the skin permeability of VC [23,24]. However, despite its low skin permeability, VC is still widely used as an active ingredient for quasi-drugs, a category regulated in Japan for functional cosmetics, with the expectation of whitening and antioxidant effects. Another water-soluble vitamin, pyridoxine (VB_6_) (basic compound; *M.W.* 169.18; log (*K_ow_* − 0.77)) (II in Figure 1), is also used in quasi-drugs with the expectation of a moisturizing effect. VB_6_ also has low skin permeability. Both of these compounds are expected to have higher efficacy if their skin permeability is improved and are also expected to lead to reduced dosages.

In this study, we selected VC and VB_6_, which are an anion and a cation, respectively, and prepare a mixture using the solvent evaporation method. Then, the changes in physicochemical properties and intermolecular interactions for both active ingredients in the mixture were examined to determine whether the mixture was an IL or not. Finally, the effect of IL conversion on the skin permeability of both active ingredients was evaluated.

## 2. Materials and Methods

### 2.1. Materials

VC, diethyl sebacate, and 1,3-butanediol were purchased from Tokyo Chemical Industry Co., Ltd. (Tokyo, Japan), and the base form of pyridoxine (VB_6_) and dimethyl sulfoxide-*d_6_* (DMSO-*d_6_*) were purchased from Sigma Aldrich (St. Louis, MO, USA).

### 2.2. Preparation of VC-VB6 IL

An IL containing VC and VB_6_ was prepared using a solvent evaporation method. After dissolving an equal molar of VC (1.76 g) and VB_6_ (1.69 g) in ethanol (200 mL), the solvent was distilled off using an evaporator at 40 °C for 1 h to obtain a slightly yellow and highly viscous substance containing VC and VB_6_ as constituents. Hereinafter, this will be referred to as a “highly viscous substance” until IL conversion is confirmed.

### 2.3. Differential Thermal Analysis

The melting point of the highly viscous substance was measured using a differential scanning calorimeter (DSC) (Thermo Plus EVO/DS C8230, Rigaku Corporation, Akishima, Tokyo, Japan). In addition to this highly viscous substance, VC alone, VB_6_ alone, and their physical mixture (by mixing using a mortar) were also measured for comparison. Each sample was put in an aluminum sample container (Rigaku Corporation), and the thermal behavior was measured from 10 °C to 300 °C at a heating rate of 10 °C/min.

### 2.4. X-ray Diffraction Measurement

The crystallinity of the highly viscous substance was evaluated using an X-ray diffractometer (XRD) (Mini Flex II, Rigaku Corporation). VC alone, VB_6_ alone, and the physical mixture were used for comparison. Each sample was placed on a glass sample plate (Rigaku Corporation), and the operation range and scanning speed were set to 2.0–60.0° and 3°/min, respectively.

### 2.5. Nuclear Magnetic Resonance Measurement

The intermolecular and intramolecular interactions between VC and VB_6_ in the highly viscous substance were evaluated using a nuclear magnetic resonance (NMR) apparatus (Varian 400-NMR, Agilent Technologies, Santa Clara, CA, USA). VC alone, VB_6_ alone, and their physical mixture were also used for comparison. DMSO-*d_6_* was used as a solvent, and the weight of each sample was adjusted to 10 mg.

### 2.6. Solubility Measurement in Diethyl Sebacate and 1,3-Butanediol

The highly viscous substance was dropped into diethyl sebacate and 1,3-butanediol and stored at 32 °C for over 72 h to prepare highly viscous substance-saturated 1,3-butanediol and diethyl sebacate, respectively. Then, the solubilities of VC and VB_6_ in each solvent were measured by high-performance liquid chromatography (HPLC).

### 2.7. Determination of VC and VB6

Each sample containing VC and VB_6_ was mixed with an equal volume of acetonitrile and centrifuged at 21,500× *g*, 4 °C for 10 min using a centrifuge (Himac CT15RE, Koki Holdings Co., Ltd., Tokyo, Japan). Then, 20 µL of the supernatant was injected into an HPLC. The HPLC system consisted of a pump (LC-20AD, Shimadzu Corp., Kyoto, Japan), an injector (SIL-20AC, Shimadzu Corp.), a column oven (CTO-20AC, Shimadzu Corp.), a UV-VIS detector (SPD-M20A, Shimadzu Corp.), a system controller (CBM-20AC, Shimadzu Corp.). An NH_2_ column (Inertsil^®^ NH_2_, 5 µm, 4.6 × 150 mm, GL Science, Tokyo, Japan) was used and analyzed with an analytical data processing system (LCsolution, Shimadzu Corp.). Mobile phase and wavelength for detection for VC and VB_6_ were acetonitrile:50 mM phosphoric acid *aq.* = 20:80 and 50:50 and UV 245 nm and 290 nm, respectively.

### 2.8. Animals

Male hairless rats (WBN/Ila-Ht), weighing about 180 g and 8 weeks old were obtained from Ishikawa Laboratory Animals (Fukaya, Saitama, Japan). The rats were housed in a room at 25 ± 2 °C and the light turned on and off every 12 h. The rats had ad libitum access to water and food (obtained from Oriental Yeast Co., Ltd., Tokyo, Japan). All animal experiments and feeding methods were approved by the Institutional Animal Care and Use Committee of Josai University (Sakado, Saitama, Japan, No. JU18003, 3 April 2018).

### 2.9. In Vitro Skin Permeation Experiment

A hairless rat was anesthetized with an intraperitoneal injection of three types of anesthesia (medetomidine hydrochloride; 0.15 mg/kg, midazolam; 2.0 mg/kg and butorphanol tartrate; 2.5 mg/kg) and killed by cervical dislocation. Then, full-thickness abdominal skin was excised from the body. The subcutaneous fat and blood vessels were carefully removed on the dermis side from the excised skin, and the skin was mounted on a vertical diffusion cell (cell volume: 6.0 mL, effective permeation area: 1.77 cm^2^) [25]. To prevent oxidative degradation of VC and to hydrate the skin, 1.0 and 6.0 mL of phosphate buffered saline (PBS) containing 0.3% homocysteine were applied to the stratum corneum and dermis side, respectively, for 1 h before the skin permeation experiment. After hydration, the PBS containing homocysteine on the stratum corneum was removed, and 1.0 mL of IL solution in 1,3-butanediol at a concentration of 200, 500, or 1000 mM was applied to the stratum corneum side (176.12 mg VC/mL and 169.18 mg VB_6_/mL at 1000 mM). The same volume of the physical mixture of VC and VB_6_ at a concentration of 200 or 1000 mM in 1,3-butanediol was also tested for comparison. These samples were suspended in 1,3-butanediol because their solubilities in 1,3-butanediol were much lower than their IL sample (see details in Results).

During the experiment, the cell was maintained at 32 °C and the inside of the receiver cell was constantly stirred magnetically with a star head type stirrer. An aliquot (0.5 mL) was sampled from the receptor compartment over time, and the same amount of PBS was replenished to the dermis each time. VC and VB6 concentrations in the sample solution were measured by HPLC. All in vitro skin permeation experiment was performed with 3 to 4 repeats.

## 3. Results

### 3.1. Effect of Ionic Liquid on the Melting Points of VC and VB6

**Figure 2:**
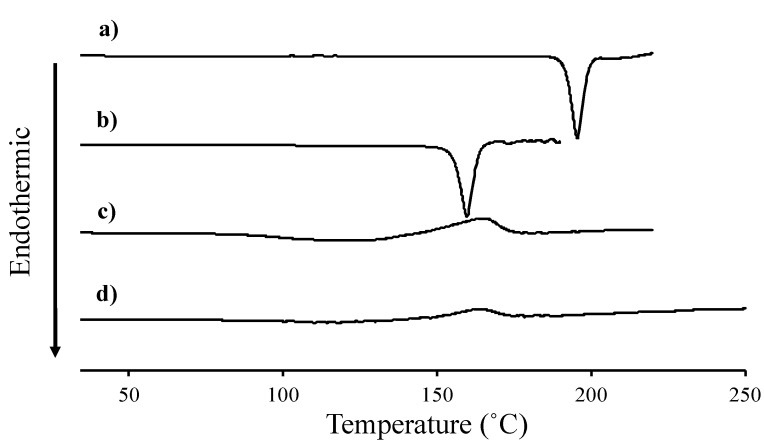
DSC thermograms of L-ascorbic acid (VC) (**a**), pyridoxine (VB_6_) (**b**), physical mixture of VC and VB6 (**c**), and their ionic liquid (IL) (**d**).

Figure 2 shows a DSC thermogram for VC and VB_6_. Four samples (original powder of VC and VB_6_, their physical mixture, and a highly viscous substance prepared in this study) were tested. The original powders of VC and VB_6_ showed endothermic peaks at 190.1 °C and 159.8 °C, respectively (Figure 2a,b). These were due to their melting. On the other hand, no clear melting points were found from room temperature to over 200 °C and a broad exothermic peak was observed at about 165 °C for the physical mixture and the highly viscous substance (Figure 2c,d).

### 3.2. Effect of Ionic Liquid on the Crystalline Properties of VC and VB6

**Figure 3:**
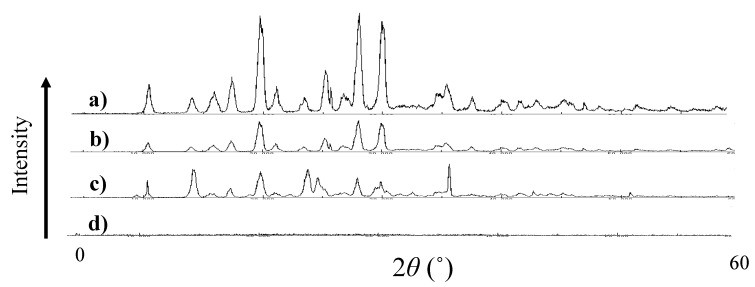
Powder X-ray diffraction patterns of VC (**a**), VB6 (**b**), physical mixture of VC and VB6 (**c**), and their IL (**d**).

Figure 3 shows the powder X-ray diffraction patterns for VC and VB_6_. Original powder of VC (a) and VB_6_ (b), their physical mixture (c), and the highly viscous substance (d) were also tested as in Figure 2. Several diffraction peaks were found in the original powders of VC and VB_6_, those derived from their crystal structures as shown in Figure 3a,b. Similar kinds of peaks were observed in the physical mixture of VC and VB_6_ (Figure 3c). Although the diffraction pattern in Figure 3c for the physical mixture was close to that in Figure 3b for VB_6_ rather than that in Figure 3a for VC and generally lower peaks compared with that for VC, no clear reason was estimated. On the other hand, no diffraction peaks were observed in the highly viscous substance (Figure 3d), suggesting that amorphization has been confirmed. It was also suggested that there was some interaction between VC and VB_6_ in the highly viscous substance prepared by solvent evaporation, which were different from the physical mixture. To clarify the difference between the physical mixture and the highly viscous substance, ^1^H NMR was then used.

### 3.3. Effect of Ionic Liquid on Intermolecular Interactions between VC and VB6

**Figure 4:**
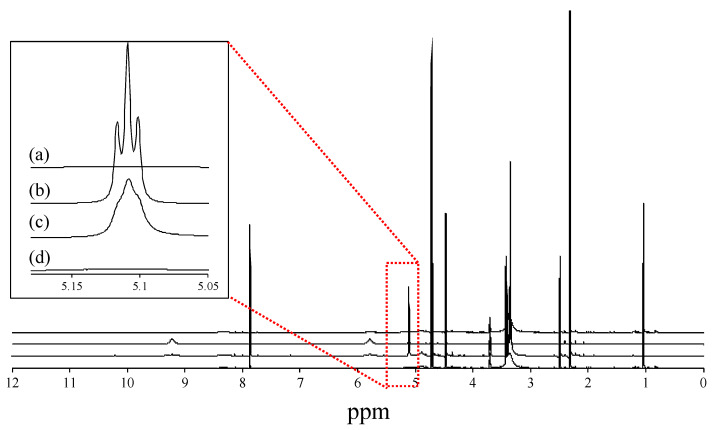
1H spectrum of VB6 (**a**), VC (**b**), physical mixture of VC and VB6 (**c**), and their IL (**d**).

Possibilities of the intermolecular interaction between VC and VB_6_ as well as and the intramolecular interaction in these compounds were evaluated for the physical mixture and the highly viscous substance using NMR. Figure 4 shows the ^1^H NMR spectra obtained by NMR measurements. Again, the original powder of VC and VB_6_, their physical mixture, and the highly viscous substance were tested as in Figure 2 and Figure 3. We paid attention only to the signal that appeared at around 5 ppm for VB_6_. This triplet signal at 5 ppm disappeared with the addition of deuterium oxide (D_2_O). Thus, this signal can be assigned at a hydroxyl proton making an intramolecular hydrogen bond (Figure 4b). A similar peak profile derived from a hydrogen bond was confirmed in the physical mixture (Figure 4c), although the signal was observed as a broad triplet, as shown in Figure 4c. However, such a peak profile disappeared in the highly viscous substance (Figure 4d). It was considered from the above data that the highly viscous substance for VC-VB_6_ prepared in this study has a different intramolecular interaction from the physical mixing treatment. Results shown in Figure 3 and Figure 4 indicated that the highly viscous substance must be an IL. Hereinafter, this substance is referred to as IL. The present experiments using ^1^H NMR spectroscopy suggested that the LC conversion eliminated intramolecular hydrogen bonds found in VB_6_. The presumed structural formula is II’ (see Figure 1) for the original powder of VB_6_ and the physical mixture of VC-VB_6_. VB_6_ had a hydrogen bond in the molecule, but it was eliminated when preparing the IL form.

### 3.4. The Solubility of VC and VB6 in Diethyl Sebacate and 1,3-Butanediol

**Figure 5:**
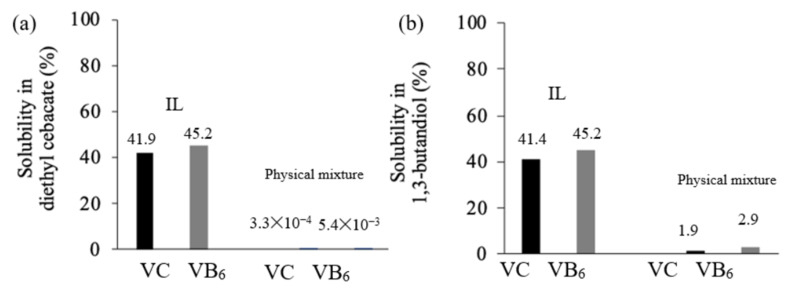
The solubility (%) of VC-VB6 from the IL and physical mixture of VC and VB6 in diethyl sebacate (**a**) or 1,3-butanediol (**b**).

Figure 5a,b show the solubility of VC and VB_6_ in diethyl sebacate and 1,3-butanediol, respectively. The physical mixture and IL for VC and VB_6_ were used as samples. The solubility of both VC and VB_6_ from their physical mixture was very low both in diethyl sebacate and 1,3-butanediol. This was as expected because these original compounds are both highly hydrophilic. The solubility of these compounds in 1,3-butanediol was higher than that in diethyl sebacate. This is due to 1,3-butanediol being more highly hydrophilic than diethyl sebacate. On the other hand, the IL form was markedly soluble both in diethyl sebacate and 1,3-butanediol. The solubility for VC and VB_6_ in diethyl sebacate was calculated as 41.9–45.2%, and that for VC and VB_6_ in 1,3-butanediol was 41.4–45.2%. The weight ratio (or molar ratio) of VC and VB_6_ in the LC form was estimated to be almost 1:1, because the molecular weights of these compounds is similar to each other. Determination of the solubility of the IL in diethyl sebacate was a little difficult because IL was not fully miscible with the solvent. Then, VC and VB_6_ concentrations in the diethyl sebacate-absorbed IL phase were determined. As explained above, the present IL showed much higher VC and VB_6_ solubility in diethyl sebacate as in 1,3-butanediol. Almost 90 g of IL dissolved in 100 g of solution.

### 3.5. Skin Permeation of VC and VB6 from 1,3-Butanediol

**Figure 6:**
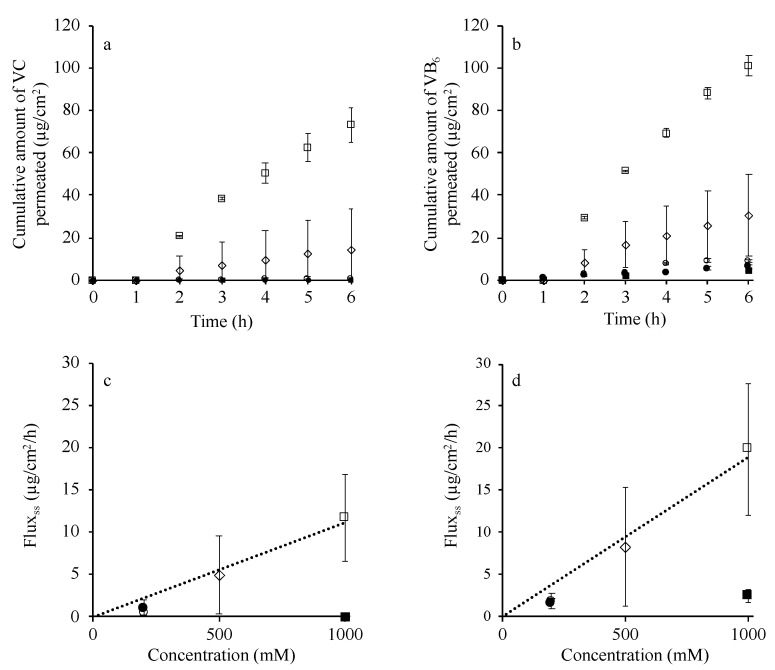
Time course of the cumulative amount of VC (**a**) and VB6 (**b**) permeated through hairless rat full-thickness skin from their 1,3-butanediol solution and the relationship between applied concentration and the calculated flux of VC (**c**) and VB6 (**d**) from this figure a and b. Symbol: ○ 200 mM IL, ◇ 500 mM IL, □ 1000 mM IL, ● 200 mM physical mixture (35.22 mg VC/mL and 33.84 VB_6_/mL), ■ 1000 mM physical mixture (176.12 mg VC/mL and 169.18 mg VB_6_/mL). Each value shows the mean ± S.D. (*n* = 3–4).

Finally, skin permeation of VC and VB_6_ was determined from the IL form presently using the original powders of VC and VB_6_. Full-thickness hairless rat abdominal skin was used in this experiment. Figure 6a and b show the cumulative amount of VC and VB_6_, respectively, permeated through rat skin from the prepared IL in 1,3-butanediol at a concentration of 200, 500, or 1000 mM. The IL solution (1.0 mL) was applied to 1.77 cm^2^ of skin, thus the applied dose of VC and VB_6_ was calculated to be 99.5 and 95.6 mg/cm^2^, respectively, in the case of the 1000 mM application. The physical mixture of VC and VB6 in 1,3-butanediol was used for comparison. Due to their low solubilities in 1,3-butanediol, the same amount of VC and VB_6_ compared to 200 mM and 1000 mM IL solutions (35.22 mg VC/mL and 33.84 VB_6_/mL at 200 mM; 176.12 mg VC/mL and 169.18 mg VB_6_/mL at 1000 mM) was applied as suspension. The solubility of VC and VB_6_ in 1.3-butanediol is 1.9% and 2.9%, as shown in Figure 5, which corresponded to about 108 and 171 mM, respectively. Thus, the physical mixture samples were suspended in 1,3-butanediol even when applied at 200 mM. All skin permeation profiles showed typical lag time and following pseudo-steady state flux. The time course after the lag time-period showed a slightly convex profile, although the reason was unclear.

Figure 6c and d show the relationship between the steady-state skin permeation rate calculated from data shown in Figure 6a and b and the applied concentration of VC and VB_6_ in 1,3-butanediol. The IL group showed a much higher cumulative amount of both VC and VB_6_ permeated through the skin than those from their physical mixture compared with each application of 200 and 1000 mM. The cumulative amount of skin permeation from the IL form increased in an application concentration-dependent manner, whereas that from the physical mixture did not. This was due to the saturated amount being applied both for the 200 and 1000 mM physical mixture [25]. Degradation of vitamin C in the donor and receiver compartments was also investigated after finishing the permeation study. No degradation was occurred during the experiment (data not shown).

## 4. Discussion

An IL is a kind of eutectic mixture and can be considered as a low melting molten salt. A eutectic mixture with a low melting point is formed when a powder composed of two or more components is mixed at a certain ratio. ILs are made of a range of compounds composed of anions and cations. Several ILs containing VC have been reported previously. Niemczak et al. prepared ILs of VC anion with dimethyldioctylammonium and didecyldimethylammonim cations [25]. They confirmed the strong influence of the cations on the stability of VC in air as well as in aqueous solutions. Additionally, the synthesized VC IL exhibited very good antibacterial and antifungal properties against different microorganisms, including pathogens.

In the present study, we planned to use an IL composed of both VC and VB_6_ to increase the skin permeation of both compounds. Among ILs reported thus far, cation moieties such as imidazolium, ammonium, and pyridinium type are well utilized. This is because these materials are relatively inexpensive and easily available. Such quaternary salts are usually prepared from a nitrogen-containing compound and an alkyl halide and are mixed with an appropriate anion to synthesize such ILs. On the other hand, in the present study, the cation part was VB_6_ and the anion was VC. An IL of VC-VB_6_ was prepared by dissolving both powders in ethanol and evaporating the solvent.

First, the physical properties of the highly viscous substance prepared in this study and the reason why we judged this substance to be an IL will be discussed. Removal of ethanol from the VC and VB_6_ ethanol solutions resulted in a slightly yellowish and highly viscous substance. DSC and X-ray diffraction of this substance were measured. As a result, the highly viscous substance did not show a clear melting point (Figure 2d) and, unlike the physical mixture, did not show crystallinity (Figure 3c,d). The DSC thermogram of the physical mixture (Figure 2c) was almost the same as that of IL (Figure 2d). It can be expected that VC and VB_6_ may react to become an IL when the temperature applied to the sample increased during the DSC measurement. Furthermore, the physical mixture itself may contain IL because both compounds may react during mixing in a mortar. However, the fact that the physical mixture was not entire IL was also evident from the fact that it has a diffraction pattern (Figure 3c).

Next, an experiment using ^1^H NMR spectroscopy was performed. As a result, it was suggested that the intramolecular hydrogen bond seen in VB_6_ disappeared in the LC conversion, although this phenomenon was not observed in the physical mixture. The presumed structure of VB_6_ is II’ shown in Figure 1 in free VB_6_ or VB_6_ in the physical mixture, and there is a hydrogen bond in the molecule, but VB_6_ in the IL seemed to exist as II without a hydrogen bond.

Lipid-soluble diethyl sebacate and water-soluble 1,3-butanediol were used as solvents in the solubility test. The logarithmic water–octanol partition coefficient, log *K_ow_*, of these solvents is 3.07 and –0.23, respectively. IL dissolved in a small amount of 1,3-butanediol, and the solubility for both VC and VB_6_ exceeded 40%. It was surprising that the combined solubility of VC and VB_6_ in 1,3-butanediol was about 87% (weight ratio). The solubility of VC in 1,3-butanediol was 41.9%, which was much higher than the advertised value (ca 25%) for the highest concentration of VC using polyethylene glycol from the patent information [26]. On the other hand, the IL prepared in this study was not freely soluble in diethyl sebacate. That is, when diethyl sebacate and the IL were mixed, a diethyl sebacate phase containing the IL and a diethyl sebacate phase containing no IL were formed. Therefore, the concentration of VC and VB_6_ in the diethyl sebacate phase containing IL was measured. As a result, the VC and VB_6_ solubility in diethyl sebacate was almost the same as that in 1,3-butanediol. The solubility of VC and VB_6_ from the physical mixture in 1,3-butanediol was low (1.9% and 2.9%, respectively).

Although the IL containing VC and VB_6_ prepared became more lipophilic than the original powders of VC and VB_6_, the log *K_ow_* was thought to be about 0. More detailed log *K_ow_* and solubility parameters of the IL should be able to be estimated using more solvents.

The solubility of the present IL showed higher solubility in the two solvents, confirming that the IL conversion increased the hydrophobicity compared with VC and VB_6_ alone. In addition, the amount of VC and VB_6_ that permeated through the skin was significantly enhanced by IL conversion compared with either drug alone or the physical mixture. When the IL was applied, the amount of skin permeation increased in a concentration- or thermodynamic activity-dependent manner (Figure 6). The relationship between the skin permeation rate (Fick’s law of diffusion) and concentration (thermodynamic activity) of applied drugs was clearly explained more than 50 years ago [27]. It was considered from these results that the permeation rate of these vitamins through the skin was increased by increasing the solubility of VC and VB_6_ in 1,3-butanediol by the present IL formulation.

In general, the IL: (1) Was non-volatile having almost no vapor pressure, (2) non-combustible and non-flammable, (3) had high thermal stability and did not decompose even at high temperatures of 300 °C or more, (4) has solvent-like properties and effectively dissolved various salts and organic compounds [28]. Therefore, the present IL is a candidate for a stable formulation of VC even in water.

## 5. Conclusions

IT this study, an IL form containing VC and VB_6_ components was prepared successfully. It was found that the IL compound arbitrarily mixed with 1,3-butanediol and dissolved at an extremely high concentration. Furthermore, it was considered that the present IL formation may be useful as a method for increasing the concentration and enhancing the skin permeation rates of both VC and VB_6_.
